# A scoping review protocol for the application of intersectionality in primary care research

**DOI:** 10.1186/s13643-025-02922-z

**Published:** 2025-09-26

**Authors:** Sean Urwin, Harriet Bullen, Saad Abbas, Pulkit Singh, Stephanie Gillibrand, Georgia Chatzi, Philip Britteon

**Affiliations:** 1 Health Organisation, Policy and Economics Group, School of Health Sciences, University of Manchester, Manchester, UK; 2 Cathie Marsh Institute for Social Research, Department of Social Statistics, School of Social Sciences, The University of Manchester, Manchester, UK

## Abstract

**Introduction:**

Past attempts to reduce inequalities in primary care have been met with mixed success. For initiatives to reduce inequalities, it is essential that they identify the most vulnerable groups in society to prevent any future exacerbation of inequalities. Intersectionality theory provides a framework to identify these groups via the exploration of how structural forms of social marginalisation interact to generate unique forms of inequalities. Despite this, little is known about the application of intersectionality theory in primary care research. To address this limitation, we propose a scoping review to comprehensively identify applications of intersectionality in the primary care inequalities literature.

**Methods:**

The scoping review will be guided by Arksey and O’Malley’s scoping review methodology framework. The review will search for studies using data to investigate intersectionalities in primary care context, using: (i) multiple electronic databases (including MEDLINE, EMBASE, ECONLIT, PsycArticles, Social Policy and Practice, and Scopus); (ii) OpenGrey to search the grey literature; and (iii) a forward and backward citation search. All authors will independently screen studies.

**Discussion:**

The proposed review will be the first to identify studies that have utilised intersectionality theory and methodologies in a primary care context. The findings will inform the design and evaluation of future primary care inequality interventions.

**Trail registration:**

Systematic review registration:

Open Science Framework https://osf.io/h9p83/

**Supplementary Information:**

The online version contains supplementary material available at 10.1186/s13643-025-02922-z.

## Introduction

### Background

Efforts to reduce inequalities in health and healthcare throughout the world have fallen short of their intended aims [1–3]. There still exist avoidable and systematic differences in the care that people receive, their opportunities to lead healthy lives, and subsequently, their underlying health [4].

A large evidence base has investigated these inequalities, analysing differences in experiences, access, quality, and use of care between patients across a range of personal, socioeconomic, and geographic characteristics [5–16]. For example, there is a 19-year gap in health life expectancy between those living in the most compared to the least deprived areas of England [17]. Despite this, individual studies have tended to analyse differences in care across a subset of socio-demographic characteristics in isolation. For example, by ethnicity [11], socio-economic status [15] or sexual orientation [5]. These studies may have misidentified how forms of structural disadvantage occur if they overlooked how different social, economic, and geographical factors combine with each other to create systems of disadvantage and discrimination [18]. This concept is referred to as intersectionality.

Intersectionality is a theoretical framework from sociology built on the premise that human experiences are jointly determined by multiple social, economic, and geographic factors, and cannot be assessed by considering these factors in isolation or independently [18]. The theory has formed the basis of numerous qualitative studies investigating identity and marginalisation [19, 20]. However, fewer studies have quantitatively estimated the extent to which inequalities may interact across different combinations of social factors [21].

Moreover, little is known about the extent and how intersectionality theory has been applied to investigate inequalities in health care, despite its potential impact [22]. Previous scoping reviews have analysed studies applying intersectionality theory across multiple disciplines [23], to assess inequalities in health outcomes [22], and in relation to informal care [24] but no studies have analysed differences in the care received by patients.

To address this gap, we propose a scoping review to determine the extent to which and how intersectionality theory has been applied to investigate inequalities in health care. Specifically, we propose to focus on primary care services. Understanding how intersectional forms of social oppression may lead to inequalities in primary care is particularly important given that primary care services are often viewed as the gatekeepers to further healthcare services, making them best placed to prevent further inequalities in the health and care system [25]. These mechanisms to impact health may operate through, but are not limited to, potential general practitioner bias [26], distance to a general practice [27], and timeliness of diagnosis [28].

By focusing on studies using an intersectional approach, our proposal also seeks to directly respond to the call from scholars to better understand how inequalities in primary care may be generated via different systemic forms of power processes [29, 30].

### Rationale

The rationale for our proposed review covers two of the four reasons for conducting a scoping review, outlined by Arksey and O’Malley [31]. First, we plan to examine the range and nature of studies using intersectionality theory to investigate the primary care inequalities literature. Second, we plan to identify gaps and limitations of the literature in relation to the use of intersectionality theory for future work which will help provide lessons for research to build on.

## Study aim

The proposed review aims to explore how intersectionality theory and methodologies have been applied to investigate inequalities in primary care services. To achieve our aim, we will first comprehensively search for and identify studies that have utilised intersectionality theory and methodologies in a primary care context. Second, we will extract key information from the included studies to provide a detailed assessment of how intersectionality theory has been applied. Third, we will propose recommendations for future primary care research on inequalities, suggesting methods through which intersectionality theory can be incorporated into the design of future studies.

## Methods and analysis

### Review framework and registration

The scoping review will follow the methodological framework set out by Arksey and O’Malley [31] and developed further by Levac et al. [32]. The review will be conducted and reported according to PRISMA-ScR guidance [33] which we have also used to guide the reporting of this protocol. The scoping review is registered with the Open Science Framework (https://osf.io/h9p83/). Our search strategy has also been reviewed by a research librarian.

### Stage 1: identifying the research question

Our proposed research question is: “How has intersectionality theory and methodology been applied to investigate inequalities in primary care services?”. We developed this primary research question using the population, context, and concept framework [34], where:

- The population will include all patients eligible to receive care and health professionals who provide primary care services including unregistered patients.
- The context will focus on the primary care setting. We plan to consider primary care provided in the community in a general practitioner setting, considering both primary care and primary health care services where the latter encompasses a broader definition beyond the traditional family doctor care [35].
- We use the Bauer et al. [23] definition of intersectionality to define the underlying concept of the research question as the investigation of inequalities across two or more patient characteristics. This definition is consistent with seminal studies on intersectionality theory [20, 36].

### Stage 2: identifying relevant studies

The databases and search terms that we propose to use are provided in Table 1. We plan to conduct the search in three parts, including published studies and the grey literature to maximise the scope of the search. Databases will be chosen based on their relevance to health research and ability to track citations. In the first part of the search, we will screen the title and abstract of all published studies in peer-reviewed academic journals. These will be obtained via an electronic search of the title, abstract, and keywords in Ovid MEDLINE, Embase, Econlit, PsycArticles, Social Policy and Practice and Scopus (all but the latter via Ovidsp). In the second part of the search, we will utilise the same methodology to identify published and unpublished grey literature studies, via an electronic search of OpenGrey. We propose to use no date restrictions during the search process, to ensure all potentially relevant studies are captured.

**Table 1:** Database, rationale, and search terms

Database	Rationale	Search syntax
Embase (Ovid)	Includes biomedicine and life science journals	1. (("primary care") OR ("primary health*") OR ("general practi*") OR (GP) OR ("family practi*") OR ("family physician*") OR ("family medic*) OR ("health practi*") [ti, ab, key] 2. (intersection*) [ti, ab, key] 3. 1 AND 2
Ovid MEDLINE	Includes a range of journals in the health policy and public health fields	^a^
Econlit (Ovid)	Includes a range of journals in the social sciences field	^a^
APA PsycArticles (Ovid)	Includes a range of psychology journals	^a^
Social policy and practice (Ovid)	Includes social care journals	^a^
Scopus	Multidisciplinary nature including medicine and social science fields	1. TITLE-ABS-KEY(("primary care") OR ("primary health*") OR ("general practi*") OR (GP) OR ("family practi*") OR ("family physician*") OR ("family medic*) OR ("health practi*")) 2. TITLE-ABS-KEY (intersection*) 3. 1 AND 2

^a^Same search syntax to that presented in the first row

We plan to use a range of primary care search terms, utilizing the search filter specified by Gill et al. [37]. These terms are presented in Table 1. We will use the Boolean operators “AND” and “OR” for search strings.

To assess the feasibility of these search terms, we have conducted a preliminary search in Ovid across five databases. We present the search results in Appendix 1: Table A1 which resulted in 899 abstracts. Our proposed review plans to extend this search.

We propose the following four-part inclusion criteria listed in Table 2. First, we plan to only include studies focusing on primary care or primary health care services, defined as care delivered in a GP practice or equivalent. Second, we propose to only include studies investigating inequalities in primary care using an intersectionality approach. To do so, we will use the definition of intersectionality adopted by Bauer et al. [23] that defines intersectionality as the investigation of two or more patient characteristics. Third, we will only include empirical studies using quantitative, qualitative or mixed methods. In doing so, we will exclude theoretical studies and commentary pieces. These types of studies do not align with the objectives of the scoping review which seeks to investigate the application of intersectionality in primary care and its practical implications, rather than generate theoretical hypotheses. Our search will have no publication date restriction, however, due to resource constraints, we will only include studies written in English.

**Table 2:** Proposed inclusion criteria

Inclusion criteria:	
• Studies in a primary care or primary health care context • Studies that consider intersections of their analysed sample or population • Studies using a qualitative, quantitative or mixed-methods design (excl. theoretical studies or commentary pieces) • Studies written in English	

### Stage 3: study selection

Following the search, we propose to collate and upload all identified citations into Zotero (v6) and remove duplicates. These will then be exported into Microsoft Excel to undergo an initial screening test.

**Fig. 1:**
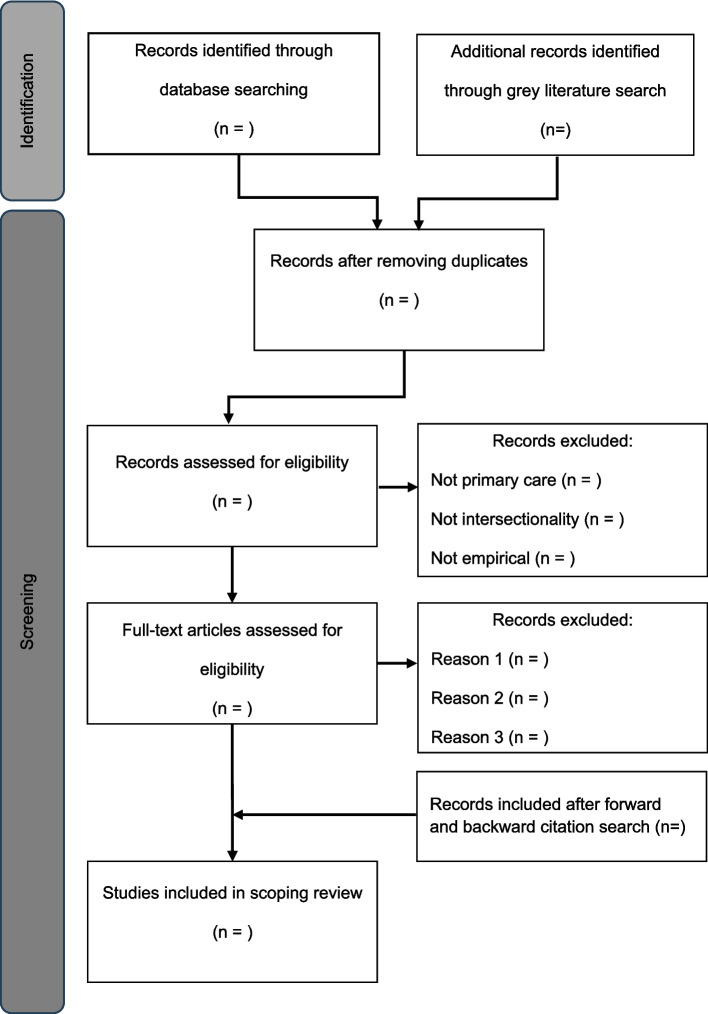
Example study selection flow chart

Titles and abstracts will be screened independently by five reviewers for assessment against the inclusion criteria. Two reviewers will conduct a 10% check of the searched studies. The group will meet to discuss the included studies and resolve any disagreements. We will then perform a forward and backwards citation search of the included studies, to identify any overlooked literature. We will record the number of studies included and excluded in the template provided in Fig. 1. The results of the search and the study inclusion process will be reported in full in the final scoping review.

### Stage 4: charting the data

We propose to extract the following information listed in the data extraction form in Table 3. One reviewer will extract information from the included papers.

**Table 3:** Proposed data extraction form

Dimensions	Details	Reason (if applicable)
General information	Authors, year, and country of study	To examine the geographical spread and development over time of the literature
Study Aim	Stated aim of the study and if intersectionality is explicitly part of this(these) aims	Identify if intersectionality is a primary aim of the study
Study participants	Patients or healthcare professionals and sample size	To examine if intersectionality has been applied to a patient or workforce primary care perspective and the sample size of these
Dataset and study design	Quantitative, qualitative or mixed-methods	Identify the type of study and the specific dataset
Data analysis method	e.g. Descriptive, regression, MADIHA, or random forest	Identify the specific method within quantitative, qualitative, or mixed-method study design
Outcomes	Type of outcome relating to primary care	Identify what type of outcome intersectionality analysis has been applied to
Intersections considered and the number of intersections	Social groups analysed	To understand the breadth and depth of the intersections analysed
Type of intersectionality	Intercategorical, Intracategorical or Anti-categorical	A key categorisation of intersectionality types [38]
Results	Main findings of the study	

We plan to use the following draft data extraction form which was developed from discussion with the authorship team. This will be modified and revised as necessary during the process of extracting data from each included evidence source. Modifications will be detailed in the scoping review. If appropriate, authors of papers will be contacted to request missing or additional data, where required. Extracted data will be recorded in Microsoft Excel. The completed data extraction form will be included in the final scoping review.

We will begin by charting the data with a pilot test of ten studies using the data extraction form which is in line with current guidance [32]. Two reviewers will complete the form on the same ten studies independently to ensure consistency between reviewers. Any disagreements will be resolved through discussion with the authorship team.

### Stage 5: collating, summarising and reporting the results

We will analyse and present the extracted data in table format. We will also provide a descriptive numerical summary of the included studies and a narrative synthesis of the results. In line with most scoping reviews we will not undertake a critical appraisal. Finally, we will use the results of the scoping review to provide a summary of the implications for future primary care policy and practice.

### Patient and public involvement

There was no patient and public involvement in this protocol.

## Discussion

The exploratory nature of the scoping review means that changes to the protocol may be required. Any deviations from the protocol as the search progresses will be documented in the final review. Ethical approval is not required because this review is summarising publicly available research.

We propose to only include peer-reviewed studies, which may result in the exclusion of information from other sources. Moreover, we plan to only include studies written in English, potentially overlooking some relevant studies.

The scoping review is intended to provide a comprehensive assessment of the literature that applies intersectionality theory to studies relating to primary care inequalities. A detailed understanding of how intersectionality has been applied to primary care inequalities will help inform future research by identifying gaps in the literature and highlighting the limitations of previously applied methodologies. In turn, this research will help to inform future primary care policy, helping to design and evaluate interventions that take an intersectionality perspective to identify and improve health care outcomes amongst the most disadvantaged groups in society. The full review will be published in an academic peer-reviewed journal.

## Supplementary Information

Supplementary Material 1.

## Data Availability

All data generated or analysed during this study are included in this published article [and its supplementary information files].

## Appendix 1

**Table A1:** Preliminary Ovid dataset search

Ovid search example: Embase, MEDLINE, Econlit, APA PsycArticles, Social policy and practice	
1. (("primary care") OR ("primary health*") OR ("general practi*") OR (GP)) [ti, ab, key] {825,066} 2. (("family practi*") OR ("family physician*") OR ("family medic*) OR ("health practi*")) [ti, ab, key] {174,537} 3. 1 OR 2 {920,660} 4. (intersection*) [ti, ab, key] {62,076} 5. 3 AND 4 {899}	

## References

[1] Kingsfund. Equity and endurance: how can we tackle health inequalities this time? The King’s Fund 2022. https://www.kingsfund.org.uk/publications/how-can-we-tackle-health-inequalities (accessed June 30, 2022).

[2] El Arifeen S, Grove J, Hansen PM, Hargreaves JR, Johnson HL, Johri M, et al. Evaluating global health initiatives to improve health equity. Bull World Health Organ. 2024;102:137–9. 10.2471/BLT.23.290531. 38313152 10.2471/BLT.23.290531 PMC10835640

[3] Loder E. Progress against health inequities is slow and obstacles are numerous. BMJ. 2022;376: o470. 10.1136/bmj.o470.

[4] The Health Foundation. Health equity in England: the marmot review 10 years on - the health foundation 2020. https://www.health.org.uk/publications/reports/the-marmot-review-10-years-on (accessed June 30, 2022).

[5] Elliott MN, Kanouse DE, Burkhart Q, Abel GA, Lyratzopoulos G, Beckett MK, et al. Sexual minorities in England have poorer health and worse health care experiences: a national survey. J Gen Intern Med. 2015;30:9–16. 10.1007/s11606-014-2905-y. 25190140 10.1007/s11606-014-2905-y PMC4284269

[6] Sakellariou D, Rotarou ES. Access to healthcare for men and women with disabilities in the UK: secondary analysis of cross-sectional data. BMJ Open. 2017;7: e016614. 10.1136/bmjopen-2017-016614. 28893735 10.1136/bmjopen-2017-016614 PMC5629679

[7] Urwin S, Whittaker W. Inequalities in family practitioner use by sexual orientation: evidence from the English General Practice Patient Survey. BMJ Open. 2016;6: e011633. 10.1136/bmjopen-2016-011633. 27173816 10.1136/bmjopen-2016-011633 PMC4874176

[8] Cookson R, Propper C, Asaria M, Raine R. Socio-economic inequalities in health care in England. Fisc Stud. 2016;37:371–403. 10.1111/j.1475-5890.2016.12109.

[9] Heje HN, Vedsted P, Sokolowski I, Olesen F. Patient characteristics associated with differences in patients’ evaluation of their general practitioner. BMC Health Serv Res. 2008;8: 178. 10.1186/1472-6963-8-178. 18715502 10.1186/1472-6963-8-178 PMC2533311

[10] Lueckmann SL, Hoebel J, Roick J, Markert J, Spallek J, von dem Knesebeck O, et al. Socioeconomic inequalities in primary-care and specialist physician visits: a systematic review. Int J Equity Health. 2021;20:58. 10.1186/s12939-020-01375-1. 33568126 10.1186/s12939-020-01375-1 PMC7874661

[11] Lyratzopoulos G, Elliott M, Barbiere JM, Henderson A, Staetsky L, Paddison C, et al. Understanding ethnic and other socio-demographic differences in patient experience of primary care: evidence from the English general practice patient survey. BMJ Qual Saf. 2012;21:21–9. 10.1136/bmjqs-2011-000088. 21900695 10.1136/bmjqs-2011-000088 PMC3240774

[12] Ogden J, Jain A. Patients’ experiences and expectations of general practice: a questionnaire study of differences by ethnic group. Br J Gen Pract. 2005;55:351–6. 15904553 PMC1463157

[13] Nielsen SS, Krasnik A. Poorer self-perceived health among migrants and ethnic minorities versus the majority population in Europe: a systematic review. Int J Public Health. 2010;55:357–71. 10.1007/s00038-010-0145-4. 20437193 10.1007/s00038-010-0145-4

[14] Salisbury C, Wallace M, Montgomery AA. Patients’ experience and satisfaction in primary care: secondary analysis using multilevel modelling. BMJ. 2010;341: c5004. 10.1136/bmj.c5004. 20940212 10.1136/bmj.c5004 PMC2954274

[15] Saunders CL, Flynn S, Massou E, Lyratzopoulos G, Abel G, Burt J. Sociodemographic inequalities in patients’ experiences of primary care: an analysis of the general practice patient survey in England between 2011 and 2017. J Health Serv Res Policy. 2021;26:198–207. 10.1177/1355819620986814. 33517786 10.1177/1355819620986814 PMC8182330

[16] Thomas GPA, Saunders CL, Roland MO, Paddison CAM. Informal carers’ health-related quality of life and patient experience in primary care: evidence from 195,364 carers in England responding to a national survey. BMC Fam Pract. 2015;16:62. 10.1186/s12875-015-0277-y. 25975608 10.1186/s12875-015-0277-y PMC4446949

[17] The Health Foundation. Inequalities in life expectancy and healthy life expectancy | The Health Foundation 2025. https://www.health.org.uk/evidence-hub/health-inequalities/inequalities-in-life-expectancy-and-healthy-life-expectancy (accessed July 3, 2025).

[18] Bauer GR. Incorporating intersectionality theory into population health research methodology: challenges and the potential to advance health equity. Soc Sci Med. 2014;110:10–7. 10.1016/j.socscimed.2014.03.022. 24704889 10.1016/j.socscimed.2014.03.022

[19] Bowleg L. The problem with the phrase women and minorities: intersectionality—an important theoretical framework for public health. Am J Public Health. 2012;102:1267–73. 10.2105/AJPH.2012.300750. 22594719 10.2105/AJPH.2012.300750 PMC3477987

[20] Nash JC. Re-thinking intersectionality. Feminist Rev. 2008. 10.1057/fr.2008.4.

[21] Richman LS, Zucker AN. Quantifying intersectionality: an important advancement for health inequality research. Soc Sci Med. 2019;226:246–8. 10.1016/j.socscimed.2019.01.036. 30733077 10.1016/j.socscimed.2019.01.036

[22] Harari L, Lee C. Intersectionality in quantitative health disparities research: a systematic review of challenges and limitations in empirical studies. Soc Sci Med. 2021;277: 113876. 10.1016/j.socscimed.2021.113876. 33866085 10.1016/j.socscimed.2021.113876 PMC8119321

[23] Bauer GR, Churchill SM, Mahendran M, Walwyn C, Lizotte D, Villa-Rueda AA. Intersectionality in quantitative research: a systematic review of its emergence and applications of theory and methods. SSM - Population Health. 2021;14: 100798. 10.1016/j.ssmph.2021.100798. 33997247 10.1016/j.ssmph.2021.100798 PMC8095182

[24] Hengelaar AH, Wittenberg Y, Kwekkeboom R, Van Hartingsveldt M, Verdonk P. Intersectionality in informal care research: a scoping review. Scand J Public Health. 2023;51:106–24. 10.1177/14034948211027816. 34232094 10.1177/14034948211027816 PMC9903248

[25] Hutt P, Gilmour S. Tackling inequalities in General Practice. The King’s Fund; 2010.

[26] Chapman EN, Kaatz A, Carnes M. Physicians and implicit bias: how doctors may unwittingly perpetuate health care disparities. J Gen Intern Med. 2013;28:1504–10. 10.1007/s11606-013-2441-1. 23576243 10.1007/s11606-013-2441-1 PMC3797360

[27] Todd A, Copeland A, Husband A, Kasim A, Bambra C. Access all areas? An area-level analysis of accessibility to general practice and community pharmacy services in England by urbanity and social deprivation. BMJ Open. 2015;5: e007328. 10.1136/bmjopen-2014-007328. 25956762 10.1136/bmjopen-2014-007328 PMC4431167

[28] Martins T, Hamilton W, Ukoumunne OC. Ethnic inequalities in time to diagnosis of cancer: a systematic review. BMC Fam Pract. 2013;14:197. 10.1186/1471-2296-14-197. 24359157 10.1186/1471-2296-14-197 PMC3878039

[29] Samra R, Hankivsky O. Adopting an intersectionality framework to address power and equity in medicine. Lancet. 2021;397:857–9. 10.1016/S0140-6736(20)32513-7. 33357466 10.1016/S0140-6736(20)32513-7 PMC9752210

[30] Mothupi M, Dasgupta J, Jebeli SSH, Stevenson J, Berdichevsky K, Vong S, et al. Using an intersectionality approach to transform health services for overlooked healthcare users and workers after COVID-19. BMJ. 2023;381: e072243. 10.1136/bmj-2022-072243. 37286226 10.1136/bmj-2022-072243 PMC10242615

[31] Arksey H, O’Malley L. Scoping studies: towards a methodological framework. Int J Soc Res Methodol. 2005;8:19–32. 10.1080/1364557032000119616.

[32] Levac D, Colquhoun H, O’Brien KK. Scoping studies: advancing the methodology. Implement Sci. 2010;5:69. 10.1186/1748-5908-5-69. 20854677 10.1186/1748-5908-5-69 PMC2954944

[33] Tricco AC, Lillie E, Zarin W, O’Brien KK, Colquhoun H, Levac D, et al. PRISMA extension for scoping reviews (PRISMA-ScR): checklist and explanation. Ann Intern Med. 2018;169:467–73. 10.7326/M18-0850. 30178033 10.7326/M18-0850

[34] Peters MDJ, Godfrey C, McInerney P, Khalil H, Larsen P, Marnie C, et al. Best practice guidance and reporting items for the development of scoping review protocols. JBI Evid Synth. 2022;20:953. 10.11124/JBIES-21-00242. 35102103 10.11124/JBIES-21-00242

[35] Muldoon LK, Hogg WE, Levitt M. Primary care (PC) and primary health care (PHC). What is the difference? Can J Public Health. 2006;97:409–11. 10.1007/BF03405354. 17120883 10.1007/BF03405354 PMC6976192

[36] Crenshaw K. Mapping the margins: intersectionality, identity politics, and violence against women of color. Stanford Law Rev. 1991;43:1241–99. 10.2307/1229039.

[37] Gill PJ, Roberts NW, Wang KY, Heneghan C. Development of a search filter for identifying studies completed in primary care. Fam Pract. 2014;31:739–45. 10.1093/fampra/cmu066. 25326923 10.1093/fampra/cmu066

[38] McCall L. The complexity of intersectionality. Signs. 2005;30:1771–800. 10.1086/426800.

